# Oocytes Quality Assessment—The Current Insight: A Systematic Review

**DOI:** 10.3390/biology13120978

**Published:** 2024-11-26

**Authors:** Mohd Faizal Ahmad, Marjanu Hikmah Elias, Norazilah Mat Jin, Muhammad Azrai Abu, Saiful Effendi Syafruddin, Ani Amelia Zainuddin, Nao Suzuki, Abdul Kadir Abdul Karim

**Affiliations:** 1 Advanced Reproductive Centre (ARC) HCTM UKM, Department of Obstetrics & Gynecology, Faculty of Medicine, National University of Malaysia, Jalan Yaacob Latiff, Cheras, Kuala Lumpur 56000, Malaysia; drmohdfaizal@ukm.edu.my (M.F.A.); azraiabu1983@gmail.com (M.A.A.); aniameliaz71@gmail.com (A.A.Z.); 2 Faculty of Medicine & Health Sciences, Universiti Sains Islam Malaysia, Nilai 71800, Malaysia; marjanuhikmah@usim.edu.my; 3 Department of Obstetrics & Gynecology, Faculty of Medicine, Universiti Teknologi MARA, Sungai Buloh Campus, Selangor Branch, Jalan Hospital, Sungai Buloh 47000, Malaysia; drnorazilah@yahoo.com; 4 Medical Molecular Biology Institute, National University of Malaysia, Jalan Yaacob Latiff, Bandar Tun Razak, Kuala Lumpur 56000, Malaysia; effendisy@ppukm.ukm.edu.my; 5 Department of Obstetrics & Gynecology, St Marianna School of Medicine, Kawasaki 216-0015, Kanagawa, Japan; nao@marianna-u.ac.jp

**Keywords:** oocytes quality assessment, oocytes morphology, in vitro fertilization (IVF)

## Abstract

An overall in vitro fertilization (IVF) outcome is dynamic. Good oocyte quality is important for achieving a better IVF pregnancy. Various methods have been used for OQ assessment (OQA). However, due to inconclusive evidence, one method’s superiority has not been proposed over others. Thus, OQA should be consolidated based on the current evidence for this matter. Our study aims to summarize the current OQA used and compare their recommended methods. Our results revealed that the optimal OQA for cost-effectiveness and clinical implementation is still via morphological assessment. The current method most opted for is total oocyte scoring. Although other methods—follicular fluid evaluation, mitochondria spindle assessment, or live zona imaging—can be applied with better precision, due to cost, additional tools, and expertise, and they should cater to research purposes and selected centers. Our results added value as a current strategy for optimizing OQA in IVF practice, aiming at cost-effectiveness and reproducibility for better clinical implementation.

## Simple Summary

Oocyte quality is essential in reflecting the overall IVF outcome. Thus, evaluating its quality is vital as a precursor to improving the overall cycle performance in IVF centers. To date, various methods are used for oocyte quality measures, but they need to be standardized depending on the tool and advancement of each center. The optimal reproducibility and standard measures of the oocyte quality method still need to be more conclusive. Hence, we elaborated via systematic review on the current technique of oocyte quality assessment that has been implemented worldwide. Our outcome revealed that morphological assessment via oocyte quality scoring provided an excellent, cost-effective prediction and was easily implemented even in low-resource centers. However, the learning curve is considered challenging in reducing bias; thus, a proper training protocol should be advocated. Our results added value as a current insight into oocyte quality assessment for optimizing the IVF outcome worldwide.

## 1. Introduction

The Total Fertility Rate (TFR) is alarming worldwide. Thus, the Assisted Reproductive Technique (ART) field, mainly in vitro fertilization (IVF), has become significantly important in this matter [1]. The evolution of the ART procedure has been reported throughout the years, starting with intracytoplasmic insemination (ICSI) to in vitro maturation (IVM), and Artificial Oocytes Activation (AOA), as well as in vitro activation (IVA) [2,3]. In addition to these, most of the centers utilize standard Controlled Ovarian Stimulation (COS) with the standard practice of IVF laboratories based on global recommendation. Nevertheless, the overall IVF outcome remains low despite the advances in IVF techniques worldwide [4]. Therefore, the molecular assessment of oocyte quality is vital in predicting IVF outcome. Established evidence has reported that oocytes contain various cellular components that reflect the quality that can determine potential embryo development [5]. To date, most oocytes have been evaluated morphologically under the standard microscopic with standard scoring for oocyte quality assessment (OQA) [6,7]. Previously, the OQA via morphological assessment (MA) was initially performed using five standard parameters, mainly the size of the oocytes, the color and integrity of the cytoplasm, the intactness of the zona pellucida (ZP), the polar body [8] morphology, and the presence of vacuoles [9,10]. The parameters were evaluated with scores of 0 for regular parametes and −1 for abnormal parameters, with scores of 0 considered to be the best OQ [10,11]. Subsequently, OQA via MA was further elaborated by Lazzoroni-Tealdi et al. in 2015 with the introduction of Total Oocyte Scoring [8] utilizing six parameters with additional perivitelline space (PVS) as compared to OQA scoring [12]. Otherwise, a TOS score of +6 is considered the best OQ. Since then, various scoring methods have emerged as modifications of the parameters have been proposed based on new evidence or based on the suitability of local practice [13]. Regarding MA, various studies have reported the pitfalls of this method, mainly its non-objective assessment with possible operator bias and the need for trained personnel for proper evaluation and reproducibility [14,15]. Thus, additional measures were then added to consolidate the OQA methods utilizing different cells—granulosa cells (GCs), or cumulus cells (CCs), and oocytes follicular material—follicular fluid (FF) via different assay or method, and specific tools of evaluation such as a polarized microscope for the assessment of meiotic spindles [14,16,17,18]. Despite the various methods implemented for OQA, there is still inconclusive evidence on which method is considered acceptable to be implemented as standard care in reproductive centers. Therefore, our review aims to delineate the various methods of OQA that are currently available and subsequently compare the feasibility and reproducibility in assessing overall oocyte quality. Our review also aims to consolidate the standard yet simplified method that is potentially suitable as the OQA method to be implemented in current clinical practice, aiming to improve overall IVF outcomes.

## 2. Materials and Methods

### 2.1. Protocol Registration

This systematic review was part of our research funded by the Fundamental Research Grant Scheme (FRGS), awarded by the Ministry of Higher Education of Malaysia with the title of “Elucidating the GREM1, HAS2 and PTGS2 gene expression as oocytes development competency markers among women with poor ovarian reserve following the in vitro maturation (IVM)”. The grant number was FRGS/1/2021/SKK01/UKM/02/1. In addition to that, our review also adhered to the standard guideline protocol based on the preferred reporting items for systematic reviews and meta-analyses (PRISMA) [19]. It was registered in the international database of prospectively registered systematic reviews known as PROSPERO with the registration number CRD42024561572 [20].

### 2.2. Information Source and Search Strategy

The publications within 20 years (2004–2024) were searched using the following keywords in Pub Med, SCOPUS, EBSCOHost, and Science Direct [All Fields]: ‘In vitro fertilization’ OR ‘In vitro fertilization’ OR IVF OR ‘fertilization in vitro’ AND ‘oocytes assessment’ OR ‘oocytes scoring’ OR ‘oocytes grading’ OR ‘oocytes morphology classification’ AND ‘oocytes quality’. Subsequently, all the included studies’ references were screened for duplication using EndNote^®^ version 20.0.1. The search was improved by manually using the reference lists from selected articles.

### 2.3. Study Selection, Data Extraction, and Risk of Bias Assessment

Based on an initial search, four authors (A.M.F., M.H.I., M.J.N., and .K.) screened all the titles and abstracts of the potential manuscripts. The selection criteria included manuscripts published in English from January 2004 to July 2024, with a method of oocyte quality assessment based on oocyte quality scoring, oocyte grading, or oocyte morphology classification in the human oocyte cohort. Following the title and abstract screening, full-text screening was conducted, excluding manuscripts that had no proper oocyte quality [21] assessment, a combination of the cumulus–oocytes complex (COC) as OQ, a non-English language, any case reports or review, and articles involving non-human subjects. The remaining potential manuscripts were then independently reviewed. The final selected manuscripts provided a detailed study design, focusing on a oocyte quality [21] assessment. The conflicts in selection among the authors were resolved through detailed discussions and opinions provided by the fifth, sixth, and seventh authors (S.S.E, N.S., and A.A.Z.). Subsequently, the National Institutes of Health (NIH) tool for observational studies was employed to assess the quality of the selected manuscripts. This evaluation was based on 14 variables, with a scoring system of 1 for ‘yes’, 0 for ‘no’, or ‘non-applicable’ for N.A. The manuscripts were then categorized as poor (0–5), fair (6–9), or good (10–14) based on their total scores [22]. Overall, the included studies in our review achieved a mostly good score—at least 11 (Supplementary Table S1). Subsequently, the final data were extracted and organized based on the authors’ last names, the year of publication, the country, the study design, the type of cohort, and the number of samples (if applicable). Additionally, each oocyte quality assessment method was tabulated as the primary outcome.

## 3. Results

### 3.1. Systematic Review Overview

#### 3.1.1. Search Sequence and Quality Assessment

The primary search retrieved two hundred thirty studies (Supplementary Figure S1). After removing 23 duplicates, the remaining 207 articles were thoroughly screened based on our inclusion criteria. Amongst them, 136 articles were excluded, leaving 36 for full-text evaluation. After a detailed evaluation, 23 articles were discarded—one was found as a case report, one utilized animal samples, one was a study protocol without results, and one was a review. Subsequently, six studies focused on the COC appearance assessment rather than OQ, four had no proper OQ assessment method, and nine other articles focused on an oocyte maturity (OM) assessment rather than on OQ. After all, a total of 13 studies were selected for this review. All the selected articles were evaluated using the National Institutes of Health (N.I.H.) tool for observational studies to ensure quality and minimize bias. Notably, all thirteen articles obtained a minimum good score, indicating a low risk of bias (Supplementary Table S1).

#### 3.1.2. Studies Characteristics

This review included 20,066 mature oocytes—metaphase II (MII)—with most of the studies utilizing the oocytes quality scoring (OQS) method as their primary OQ assessment method [11,12,15,21,23,24]. Additional measures using follicular fluid (FF)-combined OQS were reported in two studies [16,18]. In contrast, three other studies added mitotic spindle assessment in combination with OQS [14,16,17]. Specifically, one study added protein assessment via Western blot, FF assessment, DNA fragmentation, and chromatin integrity assessment in combination with OQS [18]. Otherwise, three other studies simplified the OQS via a cytoplasm granulation assessment, an isolated mitotic spindle assessment, and live zona imaging assessment alone [25,26,27].

#### 3.1.3. Main Outcome

The main OQ assessment used OQS via a morphological parameter alone or in combination with another tool such as spindle assessment (SA), oxidation stress (OS) evaluation via FF, PBI, or DNA fragmentation, and specific protein evaluation. Otherwise, cytoplasm scoring and LZI were also reported in our review as part of the OQ assessment. All the study’s methods and outcomes are tabulated in Table 1.

- The Oocytes Quality Scoring (OQS)

**Figure 1:**
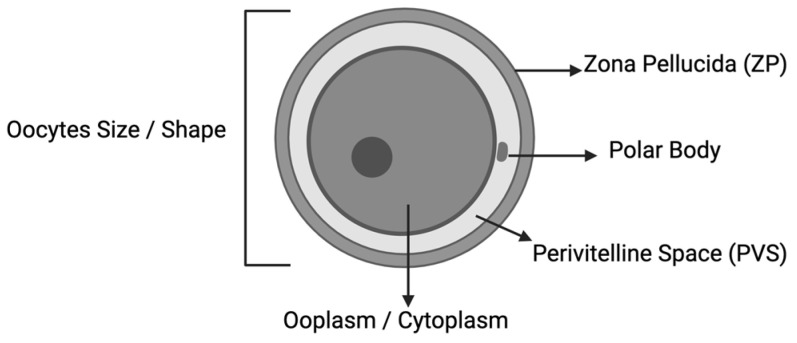
Basic oocytes morphology assessment.

**Figure 2:**
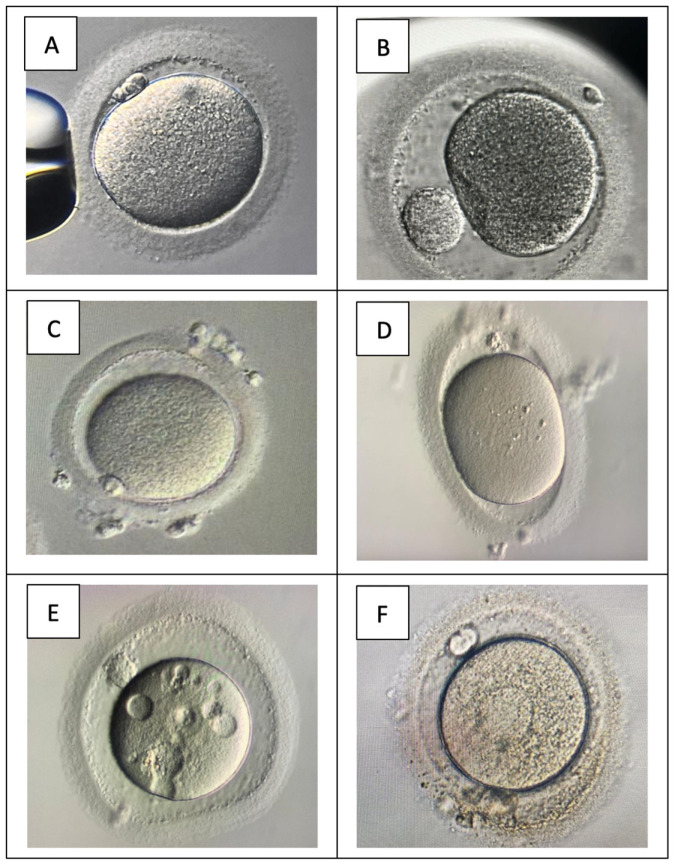
Oocytes morphology characteristic. (**A**) Normal oocytes morphology; (**B**) abnormal pb size; (**C**) abnormal PVS; (**D**) oval shape oocytes; (**E**) multiple vacuoles with abnormal PVS; (**F**) coarse ooplasm with vacuole.

Most oocyte scoring was based on five standard parameters, or Total Oocyte Scoring [8], which assesses the cytoplasmic and extracytoplasmic parameters. A total of six studies included in our review opted for morphological evaluation as OQS. Nevertheless, the OQ was related to the stress induced during the oocyte retrieval (OR) procedure, except for zona pellucida’s (ZP) intactness [30]. The sheer effect along the needle diameter raised concurrently as the higher pressure increased the follicular fluid aspiration velocity, jeopardizing the OQ outcome [31]. Meanwhile, the ZP stress-induced damage was mainly reported due to the intra-cytoplasmic sperm insemination (ICSI) procedure [32]. Moreover, the overall OQS was used to predict the embryo quality [10,15,24]. However, regardless of the initial OQS, once the oocytes were fertilized, there were no differences in potential in the embryo quality outcome despite the initial score. Most evidence concluded that the worst possible OQ ultimately depended on the fertilization ability rather than OQS itself [21]. Fundamentally, OQS is initiated following the denudation process following the oocyte retrieval (OR) procedure, preferably two to four hours later, using inverted microscopic as an essential tool. The morphological assessment of oocytes was first categorized according to the size based on the diameter. The diameter  < 120 μm was classified as small, whereas the diameter between 160 and 180 μm was classified as large, following the previously established literature [12,18]. Subsequently, the shape of the oocyte was scored as usual, likely as ovoid, uneven, or distorted. In addition, the elongated or other abnormalities in shape were regarded as distorted scores. Interestingly, few papers utilize the term ooplasm about cytoplasm for scoring. Molecularly, ooplasm is defined as the cytoplasm of the egg, including oocytes, as it is present in all eggs, unlike yolk, which can be absent in some eggs [23]. Thus, the terms cytoplasm and ooplasm are interchangeable in most scoring parameters. For these matters, our manuscript utilized ooplasm as a standard reference to oocyte cytoplasm. The ooplasm was scored as dark, granular, or vacuolated. On the other hand, the ooplasm was considered granular if granules were present at the center of the ooplasm, as established by previous evidence [25]. Nevertheless, the translucency of the ooplasm was also included in the scoring. Besides that, the perivitelline space (PVS) was then classified as usual, granular, prominent, or absent. Otherwise, the zona pelludica (ZP) was also assessed as part of the scoring mainly based on its size, as thin, regular, or thick. The ZP texture was also considered uneven, dark, granular, or regular. The final parameter—the polar body [8], was also added to the score based on its categorization as normal, small, large, flat, or fragmented [15,24]. The illustrates the morphological assessment overview (Figure 1). Overall, OQS was used as the primary outcome to compare different cohorts of women in the inclusion studies as it was considered the landmark parameter based on the established literature [24]. At the same time, only one study evaluated the OQS with the overall clinical outcome and concluded that OQS is useful clinical information in women with good prognosis [12]. The comparison of normal and abnormal oocyte morphology is illustrated in Figure 2.

- Evolution of OQS—Metaphase II Oocytes Morphology Score (MOMS)

One of the included studies further explored the standard OQS via the odds ratio utilizing significant clinical outcomes such as fertilization, 2PN, and good embryo quality, and subsequently formulated Metaphase II Oocytes Morphology Score (MOMS) scoring. Based on MOMS, once the points were combined, the lowest point was considered good oocyte quality, reflecting the possible good clinical outcome. This study also found a significant relationship between women’s age, the basal Follicular Stimulating Hormone (FSH), and the overall clinical outcome [15].

- Spindle Assessment (SA)

Besides the OQS, the meiotic spindle activity has been reported to influence overall OQ significantly [16,27]. The spindle apparatus is a vital organelle that contributes to chromosome segregation via mitotic division during the oocyte maturation phase. Therefore, there is emerging evidence regarding the role of SA as one of the critical parameters for OQ evaluation. The SA requires additional tools—a polarized imaging software module and a spider view program. Most of the centers utilize computerized systems for analysis to predict the presence or absence of MS during ICSI [17,27].

*i.*  *Spindle Assessment (SA) alone*

**Figure 3:**
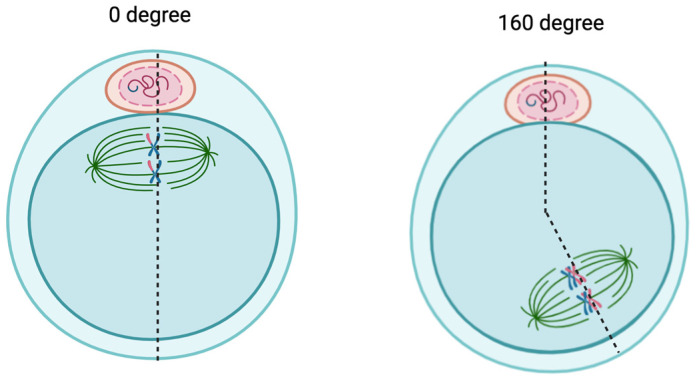
Spindle assessment (the degree measured based on PB).

Concerning SA alone, one study elaborates on using SA as a single tool in assessing the cellular level of the mitotic cell cycle with 113 oocytes. Polarization imaging software fully controlled the specialized tool for birefringence analysis with auto-calibration. In addition to that, the tool was also equipped with a motorized stage containing a fully heated ceramic plate with a glass insert in the objective pathway, allowing the rotation for oocyte monitoring and optimizing spindle mitotic band visualization for better SA. Overall, they found that spindle imaging is a technique that can improve the treatment of complex patients in assisted reproduction. It is clinically essential for OQS, enhancing SA in a synchronized oocyte maturation cohort [27] (Figure 3).

*ii.*  *Combination of OQS with Spindle Assessment (SA)*

On the other hand, to enhance SA’s role in OQ assessment, a combination of SA and OQS was performed. One of the included studies added SA to the OQ assessment. However, this study found no relationship between the presence or absence of spindle and a combination with OQS regarding the implantation rate (IR) or clinical pregnancy rates (CPR) following ICSI [14].

*iii.*  *Combination of Polar Body I Morphology (PBM) with Spindle Assessment (SA)*

On the other hand, another study combined SA with PBM, correlating the morphology of smooth, rough, fragmented, and larger PB with SA. Similarly, there was no significant relationship between the SA and PBM of the oocytes and the overall embryo quality [17].

*iv.*  *Combination of Follicular Fluid (FF) with Spindle Assessment (SA)*

The oxidation stress (OS) was considered the ultimate challenge influencing oocyte quality and jeopardizing the overall ART outcome. Therefore, the assessment of OS was mainly performed using FF which reflected the OS in the oocytes itself. One of the studies combined the FF for OS with SA to consolidate the importance of SA as part of OQS. In the cohort of high OS, mainly among women with endometriosis and PCOS, as compared to the controlled cohort, they found that there was a good correlation between SA and OS levels as reliable predictors of OQS [16].

- Cytoplasm Granulation Pattern (CGP)

The cytoplasm characteristics were reported as part of the OQ assessment, mainly in MII oocytes before ICSI. One of the studies utilized CGP in poor prognosis cohort—mainly elderly and low AMH. Overall, they concluded that the types of granulations were categorized into four groups—fine, central, dispersed, and uneven—significantly reflecting the OQ. Fine CGP represents the best OQ with the highest 2PN rate and a higher pregnancy and live birth rate compared to the others. This study suggests that CGP can be utilized as part of OQ assessment in select cohorts, mainly poor prognosis cohorts [25].

- Live Zona Imaging

**Figure 4:**
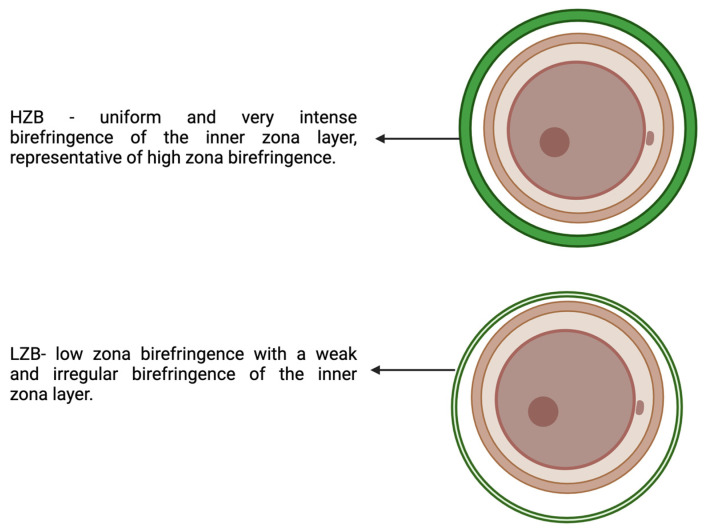
The zona birefringence.

Technically, the emergence of oocyte assessment tools—mainly the microscope—makes imaging more comprehensive and dynamic. The ability to monitor the overall quality of the cellular integrity of oocytes led to additional methods of OQ evaluation of the LZI. The autocalibration of birefringence analysis software coupled with a motorized microscope is often used for this assessment. The parameters were the intensity and uniformity of the birefringence of the inner zona layer. The High Zone Birefringence (HZB) was considered uniform in the entire cell. In contrast, the Low Zone Birefringence (LZB) was classified as uneven or low birefringence distribution in the inner zona layer (Figure 4). In one study, LZI using oocytes zona birefringence intensity was performed and revealed that embryo development was superior with HZB compared to LZB oocytes. Thus, they concluded good OQ and predictability with birefringence intensity assessment via LZI [26].

- Protein Analysis and DNA Fragmentation and Chromatin Integrity Assessment

To date, available evidence has reported that OS does interfere with the overall outcome of IVF by significantly impacting the OQ [33]. Thus, any OS assessment method can be opted to evaluate the overall OQ. Thus, in a research setting, comprehensive OS evaluation for OQ assessment can be made utilizing the COC via granulosa cells (GCs) and also FF [16]. In our review, one included study utilized this method as OQ assessment in combination with standard OQS via morphology. The OS protein markers—Hsp70, Tgf-b1, and Notch1—were used to stain the GCs for OS assessment. In addition to this, the FF was also analyzed for OS using the oxidation stress index. The index was measured using a specific spectrophotometer (Molecular 3 Multi-Mode Microplate reader) based on total absorbances of OS particles in optical density (O.D.). The low OSI was considered as ≤10 O.D., whereas the high OSI was >10 O.D. Subsequently, the TUNEL test was used to consolidate the DNA fragmentation levels by determining the chromatin integrity of cumulus cells. The positive staining revealed a dark stain, while a pale stain was considered negative staining. Overall, the study suggested that the OS within COC and FF reflected oocyte competency as they impacted the overall DNA damage and chromatin integrity as well as the expression of Hsp70 and Notch1, but not the Tgf-b1. The result also revealed that OS does affect the fertilization rates but does not affect the post-ICSI outcome, embryo quality, implantation rates, and pregnancy outcome [18].

## 4. Discussion

**Figure 5:**
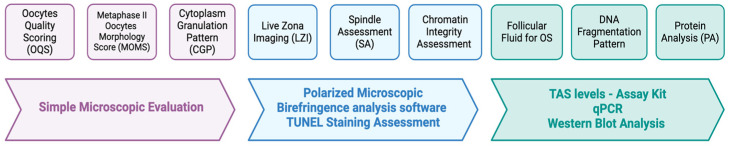
Types of OQ assessment.

The oocyte quality assessment (OQA) method is currently varied and mostly adopted based on the local protocol and availability of the expertise and advancement of laboratory tools. The current methods of OQA are shown in Figure 5. The morphology assessment (MA) is the ultimate parameter for oocytes scoring for OQA in the form of TOS [12]. Based on the latest update of the Istanbul Consensus, a revised ESHRE/ALPHA consensus on oocyte and embryo static and dynamic morphological assessment, the MA using COC appearances, oocyte characteristics of ZP, PVS, PB, size, and maturation status is the recommended parameter for OQA [8]. As elaborated in our review, most studies do opt for this method for OQA. However, the parameter of MA depends on the local protocol and reproducibility and timing of the evaluation. Most centers will adopt the MA by TOS based on these criteria. In our review, most of the included studies utilizing the latest TOS used six parameters—oocyte shape, size, ooplasm characteristic, PVS, ZP, and PB morphology—as the routine OQS. In addition to that, some included studies divided the TOS into extracytoplasmic or cytoplasmic abnormalities [15]. The OQS evolution was considered acceptable worldwide as it emerged from the fundamental TOS by Lazzaroni–Tealdi et al., aiming for better oocyte evaluation and selection [12]. The utilization of the standard tool of an inverted microscope with the power of 400× is universally practiced worldwide for the basic principle of MA for OQA. Nevertheless, we found that the OQA was coupled with other measures to consolidate the sensitivity and specificity of the OQA outcome further. To date, there is no available data regarding the sensitivity or specificity of OQA; however, evidence does show that the OQA via TOS was still predictive of clinical pregnancy rates. Previously, Lazzaroni–Tealdi et al. reported that the significant predictability using 594 oocytes correlated with the clinical outcome (OR 2.08 (95%CI 1.26 to 3.44, *p* = 0.004) [12]. However, the reproducibility was considered moderate to its operator dependence and the differentiation of subjective morphology assessment. Nevertheless, it was considered cost-effective as no additional tool was required, and it could be implemented widely. The additional value of SA via MS assessment and the utilization of FF for OS was considered a good strategy for better selection of oocytes. Nevertheless, additional tools such as polarized microscopes with additional specific molecular tests via the TUNEL test or protein analysis contribute to detailed assessment enhancing the overall assessment of OQS. However, they are only clinically applicable in some centers due to cost and expertise. Therefore, it should be offered as part of a research setting rather than a clinical service.

Our review revealed that the most straightforward approach would be the Cytoplasm Granulation Pattern (CGP), which evaluates only the ooplasm granulation pattern [25]. Although it is non-time-consuming, subjective evaluation can lead to bias. Furthermore, as only a single parameter was evaluated, the overall outcome might not truly represent overall OQ compared to the standard TOS. Therefore, further evaluation with a larger cohort should be performed before adopting this method. In addition to that, the birefringence of the inner zona layer assessment was also reported as non-time-consuming. As established, the evaluation does not require a particular device and can be performed in less than two minutes [26]. Similarly to CGP, this method is considered a subjective measurement, but requires the high specification of the microscope, and is thus limited it is clinical implementation. Highly trained personnel with proper equipment are required for this method; thus, it is not suitable for small and low-cost centers. Regarding SA, reproducibility can be achieved as the presence of MS can be reported smoothly. Nevertheless, the SA location must be based on a maneuver to ensure the correct SA is made. Based on our review, the oocytes should be rotated three times to confirm their actual absence and spindle maximum retardance should be measured along longitudinal and equatorial lines [17]. Evidence stated that the embryos resulting from oocytes with prior-to-ICSI spindle angles between 0° and 29° were associated with better fertilization and overall IVF outcome [34]. Thus, it can be technically challenging for SA and requires trained personnel and a specific microscope with polarization imaging software for evaluation. Regarding bias, the interobserver variation was reported to be low as only two oocyte SA discordants < 5%; thus, the reproducibility was considered excellent [35]. However, due to the high specification of the tools and the long learning curve, similarly to LZI, the SA for MS assessment was considered non-cost-effective and thus could not be clinically implemented widely.

Regarding FF, the measurement was mainly focusing on OS assessment. Molecularly, the FF environment reflected the overall oocyte development and maturation [36]. Established evidence reported that FF consists of particles related to receptors and their interaction during the developing and maturation phase, mainly proteins, ions, and metabolites, including the reactive oxygen species [37]. The optimal environment within FF was vital to ensure synchronization of oocyte maturation within the ovarian follicles. Thus, the mismatching of positive molecules with the reactive oxygen species led to OS, thus jeopardizing the oocyte’s quality. Therefore, FF has been utilized widely as a parameter in OQA. Nevertheless, the OQA via FF was complex, and the assessment tool varied according to the center. The chemiluminescence assay via luminometer had been reported as a method for OS measurement using FF and expressed as counted photons per second (CPS) [16]. On the other hand, the total antioxidant status (TAS) and total oxidant status were also reported as assessment tools for OS assessment via FF. In this method, spectrophotometers were used to measure both TAS and TOS, and subsequently, the oxidative stress index was calculated by dividing the TOS and TAS (OSI = TOS/TAS) [18]. Based on the complexity and level of assessment, the FF-incorporated OQA was considered time-consuming and not preferable to opt for in regard to clinical implementation. Although it helps to determine the better OQ, it could be more cost-effective and requires additional steps away from routine IVF procedures. Thus, it should be considered in a research setting.

In addition to FF, OS evidence can be consolidated with protein evaluation via immunohistochemistry (IHC). Our review included the study that extended the OS evaluation via IHC. The reported proteins were heat shock proteins (Hsp)—Hsp70, transforming growth factor-beta (Tgf-β)—Tgf-β1, and Notch1. The Hsp70 was reported as responsible for cell proliferation and development. At the same time, the Tgf-β1 was seen at the mRNA and protein level in preantral follicles which helped to regulate the cellular growth. Notch1 was also suggested as a regulator of granulosa cell proliferation and was responsible for follicular development. It was also reported that the CCs abundantly expressed the Notch genes during the folliculogenesis phase [38]. In these included studies, the OS measured the expression of three possible target proteins—Hsp70, Tgf-β, and Notch1. The Hsp70 and Notch1 were lower in the high OS group. However, there was no difference in the Tgf-β expression rates between the groups. The outcome reflected that OS leads to the suppression of Hsp70 and Notch1 expression; thus, the overall damage via OS at the cellular level could not be prevented, leading to poor OQ [18]. Molecularly, IHC requires optimizing the expression and comparing it with normal tissue; thus, the initial phase was considered lengthy. Therefore, similarly to FF evaluation, protein expression via IHC for OQA is also complex and expensive and thus should only be offered in a research setting rather than a clinical practice for now.

According to local practice, most centers have adopted OQA via MA and simplified TOS. Fundamentally, the extracytoplasmic (EC) and cytoplasmic (Cyto) characteristic assessment showed reproducibility with cost- and time-effectiveness. The strength of this method is its quick assessment of the PB, ZP, PVS, shape, and size of the oocytes followed by cytoplasmic characteristics cytoplasm, the granulated presence of vacuoles, SER clusters, or refractile bodies are standard and should be currently used as OQA methods. This is considered basic information on the overall oocyte condition that most personnel should be able to assess. A local system or protocol can also standardize the assessment steps to ensure reproducibility and reduce bias within intrapersonal scoring. Nevertheless, the emerging scoring system—Metaphase II Oocytes Morphological Score (MOMS)—can be opted for, as it simplifies the standard TOS with only two EC parameters and three Cyto parameters, as reported [15,24]. It can reduce the overall timing without reducing the overall OQA steps that are crucial in the IVF procedure. However, more data should be available for MOMS before it can be implemented as clinical practice.

As a future recommendation, all the reported OQA methods should be made available in terms of cost- and time-effectiveness. This is important to ensure that standardized evaluation can be opted for. Simplified, yet optimum evaluation is the way forward. Integrating live oocyte imaging via TOS with color contrast parameters such as birefringence analysis with additional OS analysis can be an excellent future strategy to ensure all the parameters can be assessed in one setting. The artificial intelligence (AI) algorithm of the combination assessment via simplified software can further consolidate the OQA in various reproductive centers. This technology will also help the embryologist and the clinician to obtain a better comparison of OQA outcomes based on various stimulation regimes and laboratory procedures such as ICSI, in vitro maturation (IVM), and artificial oocyte activation (AOA) in different centers, aiming to improve the overall IVF outcome worldwide.

## 5. Conclusions

The OQA should be simplified but reproducible to ensure standard measurement can be offered. Based on our review, the recommended method for now is MA via TOS with or without MOMS, which is considered acceptable for overall OQA worldwide as practically sound and clinically cost-effective with good reproducibility. Otherwise, additional measurements such as SA, FF, protein, and DNA fragmentation can be added. However, they should be offered in a research setting as they are costly, require longer timing, and require additional laboratory tools and highly skilled personnel.

## Figures and Tables

**Table 1:** Summary of the results from 13 included articles identified in a systematic review of the literature.

Author, Year	Title, Cohort, and Conclusion	Method of OQ Assessment	
Yuval Atzmon et al., 2020 [11]	Degenerative Oocytes in the Aspirated Cohort Are Not Due to the Aspirating Needle: A Prospective Randomized Pilot Study with Sibling Oocytes Cohort: 580 oocytes from 43 women 293; 17G needle group 287; 20–17G group Conclusion: Oocyte scoring was comparable between the two different needle. Cycles with degenerative oocytes in the cohort at OPU demonstrated poorer oocyte quality and decreased fertilization, regardless of the needle used.	The five standard parameters for oocyte quality are as follows: (1) size and symmetry of the perivitelline space structure; (2) color and integrity of the cytoplasm; (3) intactness of zona pellucida; (4) polar body morphology; (5) presence of vacuoles. Score: =0 if normal =−1 if abnormal All the negative parameters were summed. Scores from 0 to −5. A total score of 0 was considered the best oocyte quality. Degenerative oocytes—cytoplasm dark in color and shrunken—(DEG)	
2. Yasmine Boumerdassi et al., 2024 [23]	Impact Of Oxygen Tension During In Vitro Maturation: A Sibling-Oocyte Prospective Double-Blinded Study Cohort: In vitro maturation (IVM) culture among fertility preservation (FP)—161 IVM cycles n = 500 (group 5% O_2_), 491 (group 20% O_2_) Conclusion: Culture under low O2 tension (5% O_2_) improves oocyte morphology IVM, suggests that culture under hypoxia should be standardized.	Total oocytes quality TOS scoring—the oocytes were evaluated based on 6 parameters: (i) oocyte shape; (ii) oocyte size; (iii) ooplasm characteristics. [19] structure of the perivitelline space (PVS); (v) zona pellucida (ZP); (vi) polar body morphology. Each parameter was graded as worst (−1), average (0), or best (1) The maximal TOS of an oocyte, therefore, could be a +6, the lowest a −6	
3. Rajani et al., 2012 [16]	Assessment Of Oocyte Quality In Polycystic Ovarian Syndrome And Endometriosis By Spindle Imaging And Reactive Oxygen Species Levels In Follicular Fluid And Its Relationship With IVF-ET Outcome Cohort FF assessment; n = 637 56 women with endometriosis—Group A (n = 215); 48 women with PCOS—Group B (n = 202); 63 age-matched controls—Group C (n = 220). Cohort for MS (Meiotic Spindle); n = 637 56 women with endometriosis—Group A (with MS; 142, no MS; 73); 48 women with PCOS—Group B (with MS; 102, no MS; 101); 63 age-matched controls—Group C (with MS; 137, no MS; 83). Conclusion: Good correlation between spindle imaging and ROS levels as reliable predictors of oocyte assessment.	Follicular fluid (FF) assessment for reactive oxidation stress (ROS) FF samples were centrifuged at 300× *g* for 7 min: to remove cellular components; to clear supernatant was used for the measurement of ROS levels. 2. ROS levels in freshly aspirated FF were evaluated: Chemiluminescence assay using luminol (5-amino-2, 3-dihydro-1, 4-phthalazinedione; Sigma Chemical Co., St. Louis, MO, USA) as a probe; 400 μL of clear supernatant was placed in the cuvette of the luminometer (Berthold, Sirius Single Tube Luminometer, Model No. 0727); 10 μL of 5 mM luminol in DMSO was added to it; Each sample was scanned for 10 min; ROS values were expressed as counted photons per second (CPS). Spindle assessment Prior to the ICSI procedure The oocytes were placed on an Olympus inverted microscope with a heated stage (Tokai Hit, Thermoplate, Shizuoka-ken, Japan) at (mean ± SD) 37.0 ± 0.5 °C; Observed at 400× magnification for nuclear maturation stage; MII oocytes identified; The meiotic spindle screened using a polarization imaging software module, LC PolScope optics, and a controller (Oosight TM META Imaging System; CRI, Woburn, MA, USA); sensitivity of 0.02 nm combined with a computerized image analysis system (SpindleView software); If no MS initially were rotated a maximum of three times to confirm their true absence—with MS vs. no MS.	
4. Chamayou et al., 2006 [14]	Meiotic Spindle Presence And Oocyte Morphology Do Not Predict Clinical ICSI Outcomes: A Study Of 967 Transferred Embryos Author Links Open Overlay Panel Cohort: 404 infertile women; n = 967 oocytes Conclusion: No relationship was found between oocyte morphology or meiotic spindle presence or absence and clinical pregnancy per transfer and implantation rates after ICSI.	The parameters for oocyte quality Based on oocyte morphology ooplasm texture; perivitelline space largeness; perivitelline space granulation; absence/presence; first polar body shape. Spindle assessment The meiotic spindle screened using a polarization imaging software module, LC PolScope ProTM optics, and a controller (Spinder View: Oosight TM META Imaging System; CRI, Woburn, MA, USA; and analyzed with computerized image analysis system (SpindleView software, CRI).	
5. De Santis et al., 2005 [17]	Polar Body Morphology And Spindle Imaging As Predictors Of Oocyte Quality Cohort: 382 infertile women (n = 873 oocytes) Conclusion: PBI morphology not indicative for development potential and no significant relationship between average spindle retardance of oocytes with embryo quality.	Polar body I (PBI) morphology assessment using an inverted microscope (1 × 70 Olympus, Hamburg, Germany) Group I—normal size, smooth surface; Group II—rough surface; Group III—fragmented; Group IV—large size. Spindle assessment The meiotic spindle screened using a polarization imaging software module, LC PolScope optics, and a controller (Oosight TM META Imaging System; CRI, Woburn, MA, USA); sensitivity of 0.02 nm combined with a computerized image analysis system (SpindleView software); Oocytes were observed at equatorial plane and spindle maximum retardance was measured along two lines—longitudinal and equatorial.	
6. Jianjun Hu et al., 2021 [25]	Predictive Value Of Cytoplasmic Granulation Patterns During In Vitro Fertilization In Metaphase II Oocytes: Part I, Poor-Prognosis Patients Cohort: poor-prognosis infertile women (elderly and low AMH level, n = 2690 oocytes) Conclusion: The four distinct cytoplasmic granulation patterns in metaphase II oocytes had a predictive value for fertilization, pregnancy, and live birth outcomes in the in vitro fertilization cycles of poor-prognosis patients.	Cytoplasm granulation pattern for OQ The granulation type Fine granulation “sandy” pattern— homogeneous ooplasm with only marginally detectable small granules (<1 mm in diameter) and no granular clusters; Central granulation—“ring” pattern, made up of a single large cluster of granules located centrally; Dispersed granulation, featuring a “rocky” pattern, full of large-size granules (mostly >1 mm in diameter; Uneven granulation—mixed “rocky-sandy” pattern of large granules occupying some regions (rocky) of the ooplasm, whereas other regions showed fine (sandy) granules.	
7. Karabulut et al., 2020 [18]	Effects Of Follicular Fluid Oxidative Status On Human Mural Granulosa Cells, Oocyte Competency And ICSI Parameters Cohort: 166 infertile women (elderly and low AMH level, n = 2690 oocytes) Conclusion: Oxidative stress in FF adversely affects fertilization rates post-ICSI, but has no effect on embryo quality, pregnancy, and implantation rates. The DNA damage and chromatin integrity were increased, whereas Hsp70 and Tgf-ß decreased in the mural granulosa cells in cases of oxidative stress which may indirectly reflect the oocyte competency and may be used as biomarkers for ICSI outcome measures.	Protein analysis by immunocytochemistry and immunofluorescence Immunofluorescence analysis—to determine the expression levels by measuring relative staining intensity. The Western blot confirmed the results. To analyze the effects of oxidative stress on mural granulosa cells: Hsp70; Tgf-β1; Notch1. Follicular fluid (FF) assessment for oxidation stress (OS) Total antioxidant status (TAS) and total oxidant status [8] levels in follicular fluids were determined by using Rel assay kits (Rel Assay Diagnostics, Gaziantep, Turkey). TAS Assay Kit was used and the absorbances were measured using a spectrophotometer (Molecular Devices SpectraMax i3 Multi-Mode Microplate reader San Jose, CA, USA). The intraassay %CV values for the TAS measurement were 2.36% for the 0.50 (0.35–0.65) mmol Trolox equiv/L and 2.24% for the 2.0 (1.7–2.3) mmol Trolox equiv/L. The intraassay CV% values for the TOS measurements were 3.57% for 5.5 (3.0–8.0) μmol/L and 5.17% for 19.5 (16–23) μmol/L. Oxidative stress index [26] = total oxidant status [8]/total antioxidant level (TAS) Oocytes quality assessment Good OQ: diameter of 120–130 μm; an intact shape; a homogeneous cytoplasm; no vacuolization; no granulation; no refractile body formation; non-fragmented polar body; zona pellucida below 10 μm. Oocyte quality = (%) of good morphology oocytes/total number of mature oocytes. DNA fragmentation and chromatin integrity assessment: TUNEL test was used to evaluate DNA fragmentation levels; Toluidine blue (TB) staining was used to determine the chromatin integrity of cumulus cells; TB staining was evaluated under light a microscope as positive (dark-stained) or negative (pale-stained) by comparing dye intake.	
8. Lazzaroni et al., 2015 [12]	Oocyte Scoring Enhances Embryo-Scoring In Predicting Pregnancy Chances With IVF Where It Counts Most Cohort: 94 infertile women (n = 594 oocytes) Conclusion: Oocyte scoring thus provides useful clinical information, especially in patients with good prognosis and large numbers of high quality embryos.	Total oocytes quality [8]—TOS scoring (Lazzaroni-Tealdi et al., 2015) [12] The oocytes were evaluated based on 6 parameters: (i) Morphology Oocyte morphology was poor (dark general oocyte coloration and/or ovoid shape) = −1; If almost normal (less dark general oocyte coloration and less ovoid shape), = 0; normal = +1. (ii) Oocyte size Abnormally small or large = −1 < 120 μ or >160 μ; If the size was almost normal, i.e., did not deviate from normal by more than 10 μ, = 0.0l; Within normal range >130 μ and <150 μ, = +1; (iii) Ooplasm characteristics Very granular and/or very vacuolated and/or demonstrated several inclusions = −1; Only slightly granular and/or demonstrated only few inclusions = 0; Absence of granularity and inclusions = +1 [19] structure of the perivitelline space (PVS); Abnormally large, an absent or a very granular PVS = −1; Moderately enlarged and/or small PVS and/or a less granular PVS = 0; Normal size PVS with no granules = +1 (v) zona pellucida (ZP); Very thin or thick (<10 μ or >20 μ) = −1; Not deviate from normal by > 2 μ = 0; A normal zona (>12 μ and <18 μ) = +1. (vi) Polar body [8] morphology; Flat and/or multiple PBs, granular and/or either abnormally small or large PBs = −1; Fair but not excellent = 0; Normal size and shape = +1; Each parameter was graded as worst (−1), average (0), or best (1). The maximal TOS of an oocyte, therefore, could be a +6, the lowest a −6.	
9. Montag et al., 2006 [27]	Spindle Imaging In Human Oocytes: The Impact Of The Meiotic Cell Cycle Cohort: infertile women (n = 113 oocytes) Conclusion: Spindle imaging is a technique that can potentially improve treatment of patients in assisted reproduction that may be of clinical importance. The timing of ICSI can be fine-tuned especially in patients with difficult ovarian stimulation and/or patients who present with oocytes of different maturational stages.	Spindle assessment Non-invasively on a Nikon Eclipse TE-2000 Phihong Enterprise (Taiwan) inverted microscope The birefringence analysis including auto-calibration was fully controlled by a polarization imaging software module (OCTAX ICSI Guard^TM^; OCTAX Microscience GmbH, Herborn, Germany) implemented in a basic software system (OCTAX Eyeware^TM^); The microscope was further equipped with a motorized stage (OCTAX) containing a fully heated ceramic plate with a glass insert in the objective pathway; The temperature of the heated plate was adjusted to maintain 37.0 ± 0.5 °C in a 5 μL medium droplet in the glass bottom dish during microscopic observation; A micromanipulation system (Eppendorf, Hamburg, Germany) adapted to the microscope allowed for rotation of oocytes to optimize spindle visualization.	
10. Montag et al., 2008 [26]	Oocyte Zona Birefringence Intensity Is Associated With Embryonic Implantation Potential In ICSI Cycles Cohort: 124 infertile women (n = 1029 oocytes) Conclusion: Overall, the embryo development was superior in embryos derived from HZB oocytes. This study concludes that oocyte zona birefringence is a good selection criterion and a good predictive criterion for embryo implantation potential.	Live zone imaging Nikon Eclipse TE-2000 inverted microscope with ×10, ×20 and ×40 Hoffmann interference optics, ×20 and ×40 stain-free objectives, a circular polarization filter and liquid crystal analyser optics. The birefringence analysis—autocalibration was fully controlled by a polarization imaging software module (OCTAX ICSI Guard^TM^, OCTAX Microscience GmbH, Altdorf, Germany) with imaging software system (OCTAX Eyeware^TM^). The microscope-motorized stage (OCTAX) containing a fully heated ceramic plate with a glass insert in the objective pathway. The temperature of the heated plate maintains 37.0 ± 0.5 °C in a 5-μL medium droplet in the glass bottom dish during microscopic observation (Montag et al., 2006) [27]. Steps First polar body (PBI) by conventional light microscopy and change in focus to exclude immature oocytes (germinal vesicle, metaphase I); Individual images combining bright field (green) and birefringence (red) views were recorded online by the imaging software; Based on the intensity and uniformity of the birefringent inner zona layer, MII oocytes were classified—high zona birefringence high-intensity birefringent inner zona layer where the birefringence was mostly uniform around the entire cell were classified—(HZB) or uneven and/or low birefringence distribution around the cell were classified low zona birefringence (LZB); The classification—subjective judgment and not supported by any measuring device—allowed a rapid screening of 10 oocytes within 2 min.	
11. Rienzi et al., 2008 [15]	Significance Of Metaphase II Human Oocyte Morphology On ICSI Outcome Cohort: 516 infertile women (n = 1191 oocytes) Conclusion: A significant relationship was found between MOMS and female age, female basal FSH, and clinical outcome. The morphologic evaluation before ICSI helps to identify MII oocytes with higher developmental potential.	Oocyte morphology assessment The oocyte’s morphologic characteristics were classified as extracytoplasmic abnormalities fragmented IPB; abnormal IPB (large and/or degenerated); abnormal zona pellucida (thick and/or dark); large perivitelline space; abnormal oocyte shape (oval oocytes); Cytoplasmic abnormalities; granular cytoplasm; centrally located granular area; vacuoles; SER clusters; refractile bodies. MOMS—Metaphase II Oocytes Morphology Score (MOMS) (based on significant odds ratio (OR) to reach at least one outcome; fertilization, good PN morphology or good embryo quality) Relative mark was given to each analyzed oocyte - The oocytes with lowest score were expected → highest implantation potential	
		Parameter	Points
		Extracytoplasmic abnormalities	
		Abnormal PB I	2.0
		Large PVS	1.4
		Cytoplasmic Features	
		Granular cytoplasm	1.4
		Centrally located granular area	2.7
		Vacuoles	2.1
12. Camille-Robin et al., 2021 [24]	Impact Of Endometriosis On Oocyte Morphology In IVF-ICSI: Retrospective Study Of A Cohort Of More Than 6000 Mature Oocytes Cohort: 596 women—195 endometriosis vs. 401 control (n = 2016 MII endometriosis vs. 4073 MII control) Conclusion: Endometriosis does not have a negative impact on oocytes’ morphology in IVF-ICSI.	Oocyte morphology assessment Two oocyte morphology scores: The AOQI (Average Oocyte Quality Index) score (Sigala et al. in 2015) [28]. This score counts the number of abnormalities in oocyte morphology on 7 items; cytoplasmic granularity; zona pellucida anomaly—irregular shape or thickened zona pellucida; presence of intracytoplasmic vacuoles; material in the perivitelline space (PVS); anomaly of the first polar body (IPB); large perivitelline space and oocyte shape; The ratio of the (total number of abnormalities): (number of MII oocytes) collected. 2. The MOMS (metaphase II oocyte morphological scoring system) score was calculated for each oocyte (Rienzi et al.in 2008 [15])—attributes a different coefficient to 5 abnormalities depending on their impact on the outcome of attempts; fertilization rate; rate of zygotes obtained; rate of embryos obtained; pregnancy rates. The average MOMS scores of the oocytes collected per attempt were then calculated by the ratio of the sum of the MOMS scores of the oocytes to the number of MII oocytes collected on the attempt.	
13. Ten J et al., 2007 [21]	Donor Oocyte Dysmorphisms And Their Influence On Fertilization And Embryo Quality Cohort: 126 donor women (n = 1622 MII) Conclusion: The oocytes dysmorphisms (OD) found in the oocytes from proven fertile patients did not affect fertilization rates after ICSI. However, OD may significantly decrease (or increase) the chance of having good quality embryos.	Oocyte morphology assessment The oocyte dysmorphisms (OD) evaluated via direct comparison with normal oocytes: A. Normal oocytes clear cytoplasm with homogeneous; fine granularity; a round or ovoid first polar body; smooth surface; size within a small perivitelline space; colorless zona pellucida with regular shape. B. Oocyte dysmorphisms (OD) extracytoplasmic OD; large perivitelline space; presence of perivitelline debris; non-spherical oocyte shape; abnormal zona pellucida; fragmented/irregular first polar body; cytoplasmic OD; excessive cytoplasmic granularity; centrally located or affecting the whole gamete; presence of vacuoles and dark cytoplasm. Vacuoles that are considered fluid-filled membrane-bound cytoplasmic inclusions and can be clearly distinguished morphologically from smooth endoplasmic reticulum clusters (sERC) are implicated in implantation failure (Otsuki et al. 2004) [29]. Thus, sERC was not evaluated in this study.	

## Data Availability

Not applicable.

## Supplementary Materials

The following supporting information can be downloaded at https://www.mdpi.com/article/10.3390/biology13120978/s1. Table S1. The NIH Tool Assessment. Figure S1. PRISMA Flow Chart.
