# Supplementary figures and images for: Oocytes Quality Assessment—The Current Insight: A Systematic Review
Source: Biology (Basel). 2024 Nov 26;13(12):978. doi: 10.3390/biology13120978 (PMC11673492; doi:10.3390/biology13120978)

Supplement Figure S1. This is PRISMA Flow Diagram

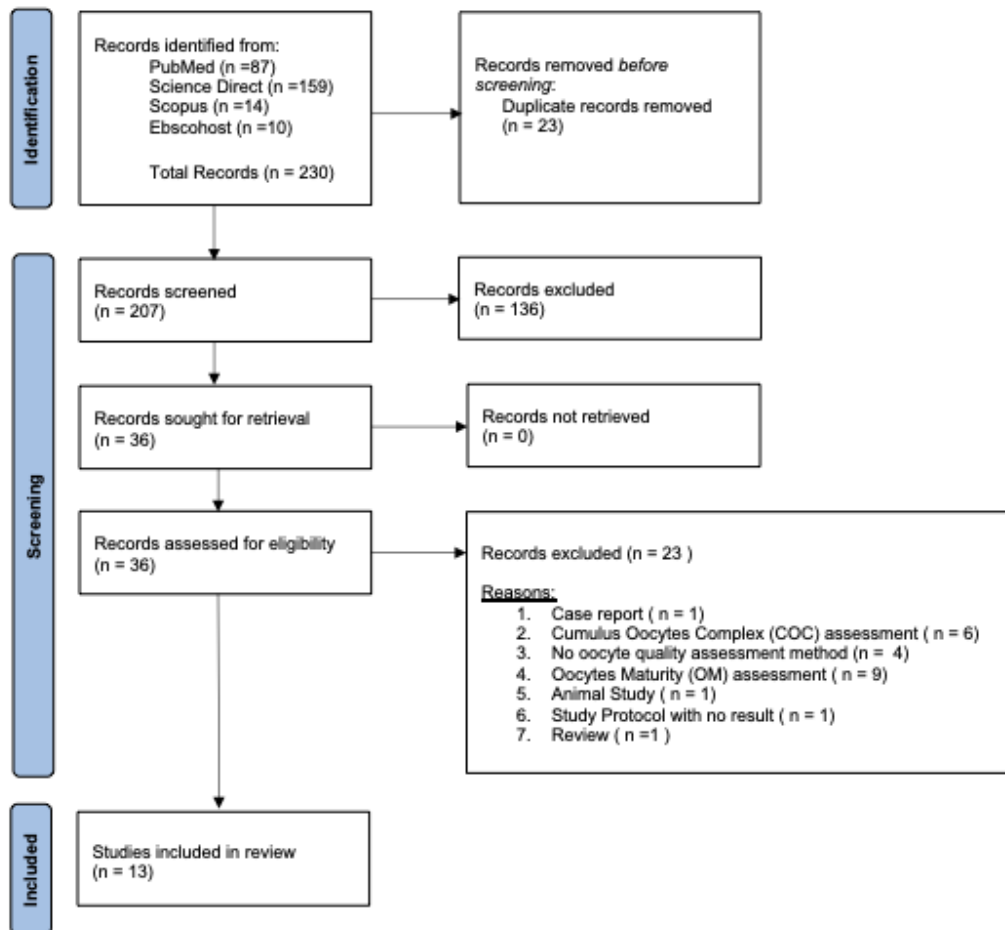

Supplement: Supplementary file 1 [file biology-13-00978-s001.zip › biology-3310124-supplementary.pdf]
